# Mesmerism

**Published:** 1844-02

**Authors:** 


### MESMERISM.

Mesmerism seems to have over-run the land. The marvellous feats, of which we heard so much from foreign parts, a few years since, are now daily performed in all the towns and villages of our country. "Such things o'ercome us like a summer's cloud," not, however, without exciting the "special wonder" of the people. A body of scientific gentlemen, in this city, performed a series of experiments, a few weeks ago, intended to test the reality of the mental sympathy, alleged to exist between the *mesmerisee* and the persons in communication with her. The report of these experiments will be published in our next number. The facts which it sets forth make a curious mass of evidence on the subject, which may enable those who are still in doubt, to form some judgment touching the pretensions of this "science." Y.

